# Surface Interaction of Ionic Liquids: Stabilization of Polyethylene Terephthalate-Degrading Enzymes in Solution

**DOI:** 10.3390/molecules27010119

**Published:** 2021-12-26

**Authors:** Zeenat Zara, Deepti Mishra, Saurabh Kumar Pandey, Eva Csefalvay, Fatemeh Fadaei, Babak Minofar, David Řeha

**Affiliations:** 1 Faculty of Science, University of South Bohemia in Ceske Budejovice, Branišovská 1760, 370 05 České Budějovice, Czech Republic; zeenaz00@prf.jcu.cz (Z.Z.); fadaei@nh.cas.cz (F.F.); 2 Laboratory of Structural Biology and Bioinformatics, Institute of Microbiology of the Czech Academy of Sciences, Zamek 136, 373 33 Nove Hrady, Czech Republic; deepti.bioinfo@gmail.com (D.M.); saurabhkpandey@gmail.com (S.K.P.); csefalvay@nh.cas.cz (E.C.); 3 Institute of Photonics and Electronics of the Czech Academy of Sciences, Chaberská 1014/57, 182 00 Praha 8-Kobylisy, Czech Republic

**Keywords:** molecular dynamics (MD) simulations, PETase, BHET, PET, ionic liquids (ILs)

## Abstract

The effect of aqueous solutions of selected ionic liquids solutions on *Ideonella sakaiensis* PETase with bis(2-hydroxyethyl) terephthalate (BHET) substrate were studied by means of molecular dynamics simulations in order to identify the possible effect of ionic liquids on the structure and dynamics of enzymatic Polyethylene terephthalate (PET) hydrolysis. The use of specific ionic liquids can potentially enhance the enzymatic hydrolyses of PET where these ionic liquids are known to partially dissolve PET. The aqueous solution of cholinium phosphate were found to have the smallest effect of the structure of PETase, and its interaction with (BHET) as substrate was comparable to that with the pure water. Thus, the cholinium phosphate was identified as possible candidate as ionic liquid co-solvent to study the enzymatic hydrolyses of PET.

## 1. Introduction

Due to the major concern on the global environmental crisis, research on how to recycle synthetic polymers has been promoted extensively for the last two decades. Among various synthetic polymers, the polyethylene terephthalate (PET), with its simple synthesis, robustness, and durability, led to a drastic increase in its industrial production; therefore, by the year 2020, production has reached more than 70 million metric tons [1,2,3]. Different chemical degradation approaches, e.g., hydrolysis, ammonolysis, aminolysis, methanolysis, and glycolysis have been introduced to recycle and remove the plastics. Nevertheless, these techniques need high temperatures and produce other environmental pollutants as byproducts.

The high solubility of PET in solvents, as the first step of recycling, is difficult; therefore, finding proper solvents for recycling and degrading is challenging. There are few solvents that were commonly used for PET solubilization, such as dichloroacetic acid [4,5] trifluoroacetic acid [6], phenol/1,1,2,2-tetrachloroethane solution [7], and chlorophenol [8]. Nonetheless, these solvents are not only costly but also toxic; therefore, it is desirable to use pretreated PET to avoid any secondary pollution in the environment.

The hydrophobic nature and crystallinity of PET are two major issues on dissolution and recycling of PET; therefore, finding environmentally friendly solvents is needed. Herein, ionic liquids (ILs) can be used to overcome this problem, as some ILs can partially change the crystalline structure of PET to an amorphous structure [9,10]. Moreover, as an alternative to traditionally used volatile organic compounds, many ionic liquids are environmentally friendly and some of them can enhance the stability and catalytic activity of certain enzymes [11,12]. ILs in general have unique features, such as strong solvent power for dissolution of organic and inorganic compounds, thermal stability, non-volatility, electrochemical stability, and low flammability [13,14]. Ionic liquids can be mixed with water at various concentrations, forming so-called hydrated ionic liquids, and hence, water and ILs act as co-solvents in the solution [15,16,17,18,19,20,21]. At the present time, ionic liquids have attracted enormous research interest due to their specific advantages and variability, which can be achieved by optimization of the choice of cation and anion combinations, influencing thermal stability, non-volatility, and many other properties [22,23]. Ionic liquids can be used for degradation of complex polymers, as was shown for cellulose, which can be utilized by ILs [24,25]. Degradation of PET in the presence of several ILs has been reported experimentally, and the results show that ILs have increased the degradation ability of PET [4,7]. The advantage of using ILs as a co-solvent over conventional catalysts, such as metal acetates, to recycle PET is that the purification of the glycolysis products is simpler. The mechanism of action of ILs is based on the changes of the highly crystalline structure of PET film into an amorphous structure (lost crystalline structure), which can be easily attacked by PETase during catalytic degradation.

One of the methods for disposal of plastic waste is biodegradation of plastic materials by enzymes [26]. Development of these environmentally friendly methods is desirable. In the case of PET, the terephthalic (TPA) moiety present in PET is hydrophobic, which makes it resistant to the bio-degradation process. In view of this, industries are focusing more on synthesizing bio-based—rather than oil-based—polymers. Herein, microorganisms play important roles in the clean-up of pollutants released by humans within the ecosystem. The process of enzymatic hydrolysis of PET films was first reported by Müller et al. (2005). The hydrolytic enzyme was purified from the culture supernatant of *T. fusca* [27]. Recently, the bacterial strain *Ideonella sakaiensis* was discovered and shown to grow on low-crystallinity PET films while using PET as a carbon source [28,29,30,31,32,33]. The enzyme, known as PETase, showed much higher depolymerization activity against PET films at the mesophilic temperature, unlike thermophilic PET-degrading enzymes tested to date. PETase works with a two-step process of degradation, at the first step, it degrades the PET into Mono-(2-hydroxyethyl) terephthalate (MHET) and then the different enzyme MHETase degrades the MHET into TPA and ethylene glycol [34]. The unique feature of PETase from *Ideonella sakaiensis* is the fact that it degrades PET film at room temperature, unlike other hydrolases, which function only at high temperatures [35,36]. This confirms the unique structural and sequence features of *Ideonella sakaiensis* PETase. Additionally, experiments reveal that *Ideonella sakaiensis* PETase exhibits the highest activity among the other thermophilic PET degrading enzymes [4,37].

Here, we report molecular dynamics (MD) simulations of *Ideonella sakaiensis* PETase with various ionic liquids in order to understand the effect of aqueous solutions of ILs on the structure of PETase and the catalytic/active site of PETase in the presence of ILs. For MD simulations, the wild-type of *Ideonella sakaiensis* PETase with ligand (bis(2-hydroxyethyl) terephthalate (BHET)) have been simulated in pure water and in different concentrations of three different ionic liquids, which were used as a solvent for dissolution of PET.

## 2. Results and Discussion

### 2.1. Root Mean Square Deviation (RMSD)

**Figure 1:**
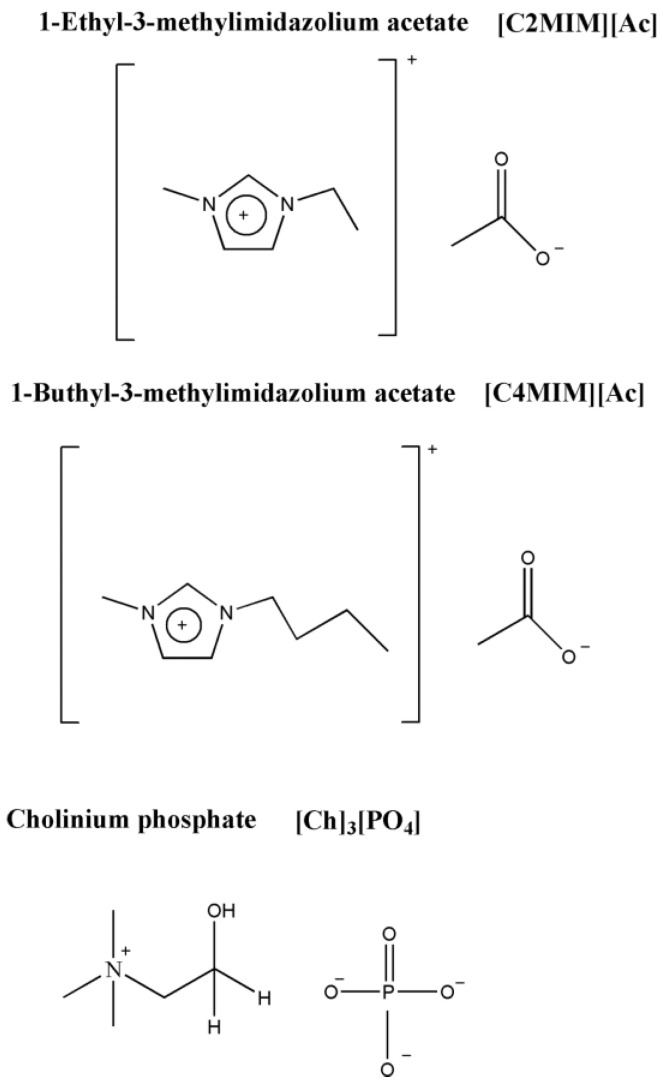
Chemical structures of 1-Ethyl-3-methylimidazolium acetate [C2MIM][Ac], 1-Butyl-3-methylimidazolium acetate [C4MIM][Ac] and Cholinium phosphate [Ch]_3_[PO_4_].

In order to investigate the effect of non-aqueous solvent on the complex structure of *Ideonella sakaiensis* PETase with ligand (BHET), we performed here MD simulations with three different ionic liquids (Figure 1) in three different concentrations (20%, 30%, 40%) by mass.

**Figure 2:**
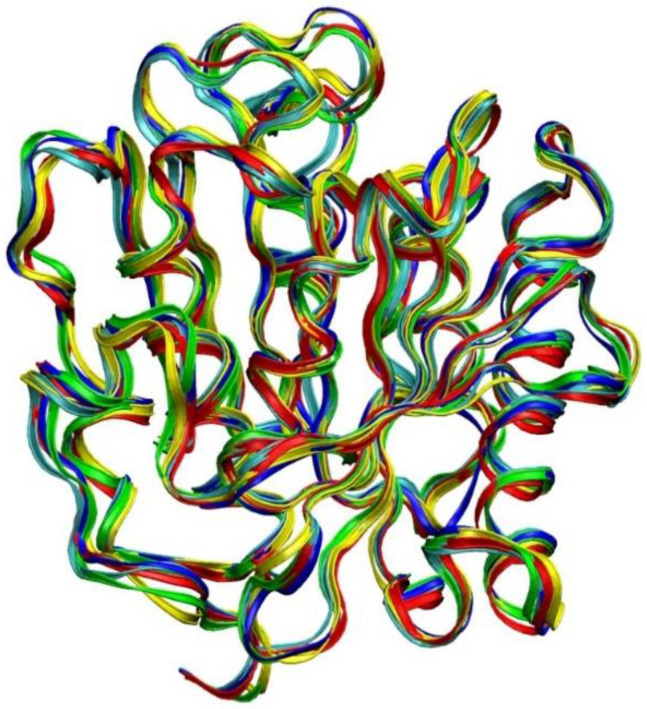
PETase backbone structure is superimposed with different solvents. The wild-type crystal structure is in blue. MD simulation for 150 ns with water as a solvent is in red. Simulation with [Ch]_3_[PO_4_], [C4MIM][Ac] and [C2MIM][Ac] is represented in yellow, green, and light blue, respectively.

The comparison of the wild-type *Ideonella sakaiensis* PETase structures in different solvents after 150 ns of MD simulation is shown in Figure 2, where the PETase backbone in different solvents was superimposed.

**Figure 3:**
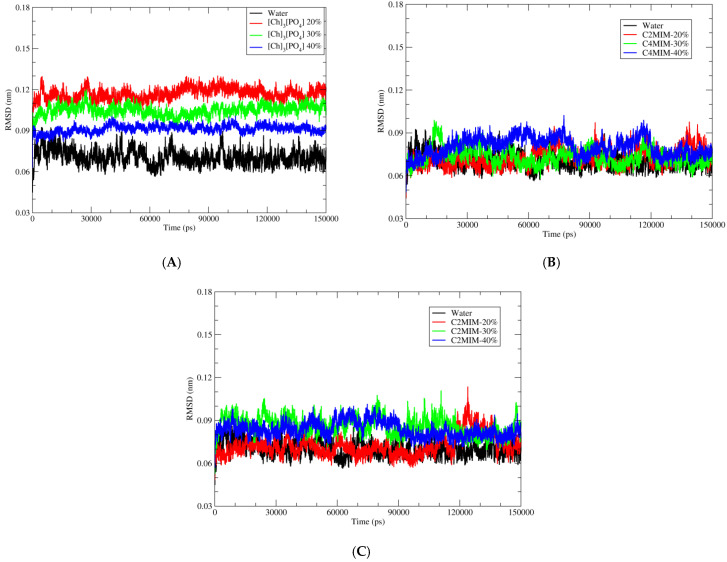
Root mean square deviation (RMSD) of Cα atoms of wild type of PETase with water and three different ionic liquids solutions. (**A**) Pure water in black; 20% of [Ch]_3_[PO_4_] in red; 30% of [Ch]_3_[PO_4_] in green and 40% of [Ch]_3_[PO_4_] in blue. (**B**) Pure water in black; 20% of [C4MIM][Ac] in red; 30% of [C4MIM][Ac] in green and 40% of [C4MIM][Ac] in blue. (**C**) Pure water in black; 20% of [C2MIM][Ac] in red; 30% of [C2MIM][Ac] in green and 40% of [C2MIM][Ac] in blue.

Simulation of the PETase complex with the ligand in water was kept as a control. Calculating the root mean square deviation (RMSD) of proteins permits the quantification of the degree of stability of the enzyme during the MD simulations. Here, in Figure 3 we have shown RMSD of backbone atoms of PETase in three different ionic liquids and in water.

As depicted in Figure 3, RMSD values for PETase in water is lower than in ionic liquids during MD simulation of all systems, indicating that PEtase structure in water is closer to the crystal structure than the structures in ionic liquids. On the other hand, the fluctuation of RMSD decreases with increased concertation of ILs, indicating more stable structures with less thermal fluctuations. Figure 3A shows the deviation of backbone atoms with respect to the reference structure (PDB code 5XJH) when inserted in water and in [Ch]_3_[PO_4_] ionic liquid with 3 different percentages: 40%, 30%, and 20%. We observed that the overall structure of PETase is not deviating much from its starting structure which implies that the chosen ionic liquids [Ch]_3_[PO_4_] would not significantly alter the enzyme structure and it is suitable for further studies on the reaction mechanism of BHET hydrolysis. We further investigate two other ionic liquids, [C4MIM][Ac] and [C2MIM][Ac]. In both [C4MIM][Ac] and [C2MIM][Ac] ionic liquids, we have not observed significant deviation of backbone atom of PETase with the addition of these ILs. The RMSD graphs for [C4MIM][Ac] and [C2MIM][Ac] is shown in Figure 3B,C, respectively.

### 2.2. Ligand RMSD

**Figure 4:**
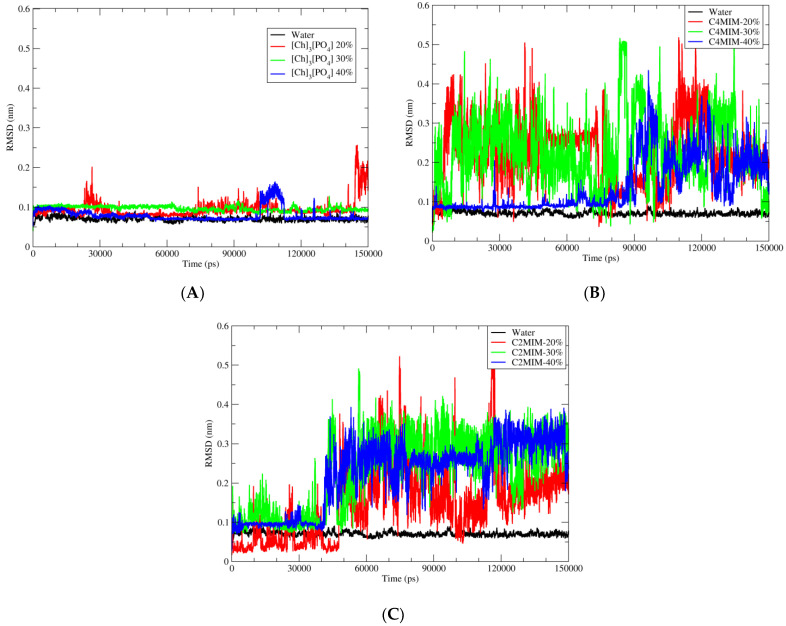
Root mean square deviation (RMSD) of BHET with respect to PETase in water and three different ionic liquids solutions. (**A**) Pure water in black; 20% of [Ch]_3_[PO_4_] in red; 30% of [Ch]_3_[PO_4_] in green and 40% of [Ch]_3_[PO_4_] in blue. (**B**) Pure water in black; 20% of [C4MIM][Ac] in red; 30% of [C4MIM][Ac] in green and 40% of [C4MIM][Ac] in blue. (**C**) Pure water in black; 20% of [C2MIM][Ac] in red; 30% of [C2MIM][Ac] in green and 40% of [C2MIM][Ac] in blue.

Analysis in the previous section showed that the enzyme structure is quite stable and not deviated much from the crystal structure after immersing into three different ionic liquid solutions. Now, the next main step is to see the reaction site of PEtase, where catalytic triad (facilitating the BHET hydrolysis) is present on the active site of the enzyme. The ligand BHET is surrounded by three catalytic amino acids, which will help BHET hydrolysis through SN-2 reaction mechanism. For this purpose, the position of the BHET ligand should be stable during MD simulations in the presence of different solvents. Therefore, we have plotted the RMSD of BHET atoms during the simulation in order to identify the stability of BHET atoms. The RMSD of the ligand, with respect to the PETase, was calculated during the simulation time of 150 ns. Here, as depicted in Figure 4A, the BHET atoms are not deviating much from their starting structure in the presence of [Ch]_3_[PO_4_].

As shown in graph Figure 4A, the ligand is quite stable in pure aqueous media and in the presence of [Ch]_3_[PO_4_] ionic liquid. However, the same stability trend has not been observed in the case of [C4MIM][Ac] or [C2MIM][Ac] ionic liquids, as shown in Figure 4B,C, respectively. A detailed inspection of the trajectory revealed that after 40 ns, the BHET molecule starts moving away from catalytic triad residue ser-131 and the BHET molecule starts bending from the central carbonyl carbon due to non-bonding interaction between two sites of BHET rings. The same trend of instability of BHET was observed for [C4MIM][Ac] after 40 ns of MD simulation, whereas the stabilization in the presence of [Ch]_3_[PO_4_] and pure water for the entire simulation indicated no significant deviation of the geometry and position of BHET molecule within PETase. Furthermore, when the concentration of ionic liquids decreased to 30% and 20%, the instability of BHET increased for [C2MIM][Ac] and [C4MIM][Ac]. Again, the molecule started to bend from the carbonyl carbon and this might be due to electrostatic interaction between two sites of BHET. The BHET structure was quite stable for the whole simulation time of 150 ns in the presence of [Ch]_3_[PO_4_] and pure water.

### 2.3. Root Mean Square Fluctuation (RMSF)

**Figure 5:**
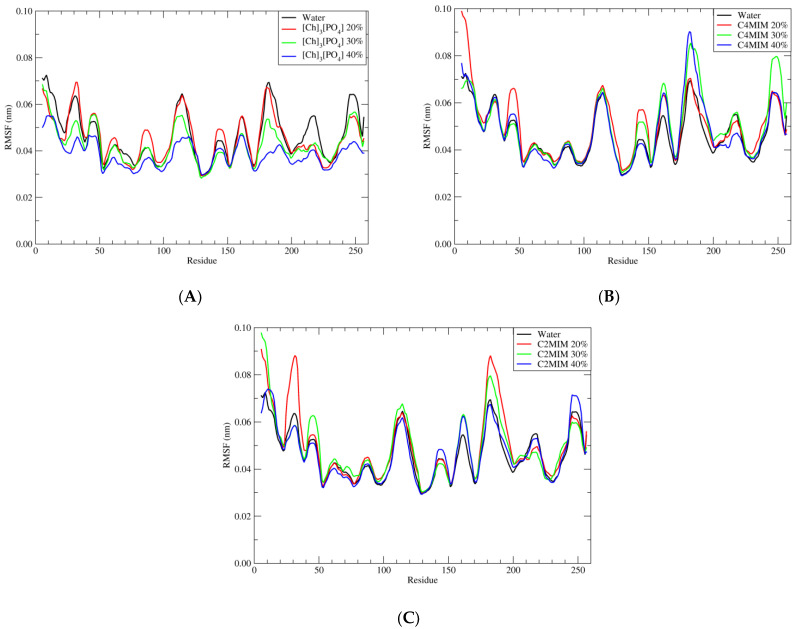
Root mean square fluctuation (RMSF) of wild-type of PETase with water and three different ionic liquids solutions in the presence of ligand BHET. (**A**) Pure water in black; 20% of [Ch]_3_[PO_4_] in red; 30% of [Ch]_3_[PO_4_] in green and 40% of [Ch]_3_[PO_4_] in blue. (**B**) Pure water in black; 20% of [C4MIM][Ac] in red; 30% of [C4MIM][Ac] in green and 40% of [C4MIM][Ac] in blue. (**C**) Pure water in black; 20% of [C2MIM][Ac] in red; 30% of [C2MIM][Ac] in green and 40% of [C2MIM][Ac] in blue.

In order to have a deep insight into structural stability, one can analyze the stability of each residue over the simulation time. The flexibility and stability of each residue is investigated by RMSF. As shown in Figure 5, the RMSF values of PETase in 40% [Ch]_3_[PO_4_] are lower than in water, which is consistent with RMSD fluctuation in Figure 3.

The residues which are fluctuating more are at residue positions 100–120 and 170–180. Among these two domains, we have found that the residue Asn-183 has the highest value of RMSF and it is the most unstable one. The same residue has the highest RMSF also in the presence of [C4MIM][Ac] and [C2MIM][Ac] ionic liquid.

To understand this further, we performed a detailed inspection of the simulations and found that [Ch]_3_[PO_4_] with 40% concentration has a strong hydrogen bond interaction between the residue Asn-183 and PO_4_^3−^. One hydrogen bond interaction between the hydrogen atom of residue Asn-183 of the PETase enzyme with the oxygen of phosphate group PO_4_^3−^ was observed. This hydrogen bond interaction was with the bond distance of 1.8 Å to 3 Å. However, we noticed that it was not stable enough over the simulation. This makes the residue Asn-183 stable relatively when we have [C4MIM][Ac] and [C2MIM][Ac] as solvents. There was no eligible entity in [C4MIM][Ac] and [C2MIM][Ac] to have an interaction with residue Asn183 and, as a result, the residue was unstable in the presence of both [C4MIM][Ac] and [C2MIM][Ac]ionic liquids; therefore, producing the higher root mean square fluctuation value.

### 2.4. Radius of Gyration

**Figure 6:**
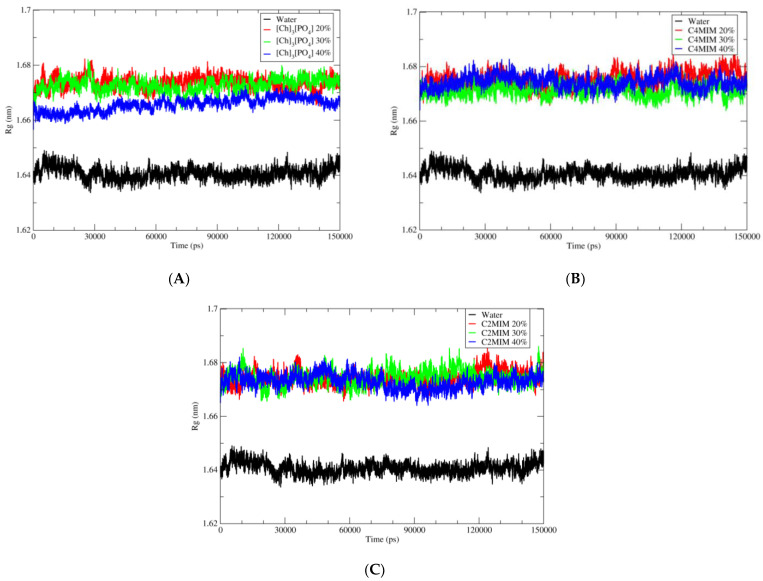
Radius of gyration (Rg) graph of wild-type PETase in complex with BHET ligand in the presence of different solvents. (**A**) Pure water in black; 20% of [Ch]_3_[PO_4_] in red; 30% of [Ch]_3_[PO_4_] in green and 40% of [Ch]_3_[PO_4_] in blue. (**B**) Pure water in black; 20% of [C4MIM][Ac] in red; 30% of [C4MIM][Ac] in green and 40% of [C4MIM][Ac] in blue. (**C**) Pure water in black; 20% of [C2MIM][Ac] in red; 30% of [C2MIM][Ac] in green and 40% of [C2MIM][Ac] in blue.

Calculating the radius of gyration of a protein is a measurement of its compactness. Thus, if a protein is stably compact, it will maintain a relatively stable value of Rg over the simulation of time. However, if a protein unfolds during the simulation of time, the R_g_ value will fluctuate. Therefore, the measurement of the compactness of protein is investigated by the radius of gyration function. The radius of gyration of each simulated system is calculated and compared between simulations in water and simulations in ionic liquid. There were no structural distortions or unfolding of the secondary structure during 150 ns simulations. However, PETase in water possesses a low radius of gyration relatively with simulated ionic liquid systems. As shown in Figure 6, the protein in ionic liquids have a radius of gyration from 1.66 to 1.68 nm, which is larger than in water, where the radius of gyration was around 1.64 nm. This indicates that the protein has a slightly less compact structure in ILs than in water. This can be explained by interactions of the IL’s ions with protein instead of the water molecules. The protein radius of gyration in ILs at different concentrations is comparable, except the [Ch]_3_[PO_4_] at 40%, where R_g_ is slightly lower at 1.66 compared with 1.67 in [C2MIM][Ac] and [C4MIM][Ac]. This is most likely due to local higher concentration of the [PO_4_], which (in comparison to then [CXMIM] and [Ac]) is less likely to disrupt local geometry of the water molecules in the vicinity of the protein (see Hofmeister series [38]).

**Figure 7:**
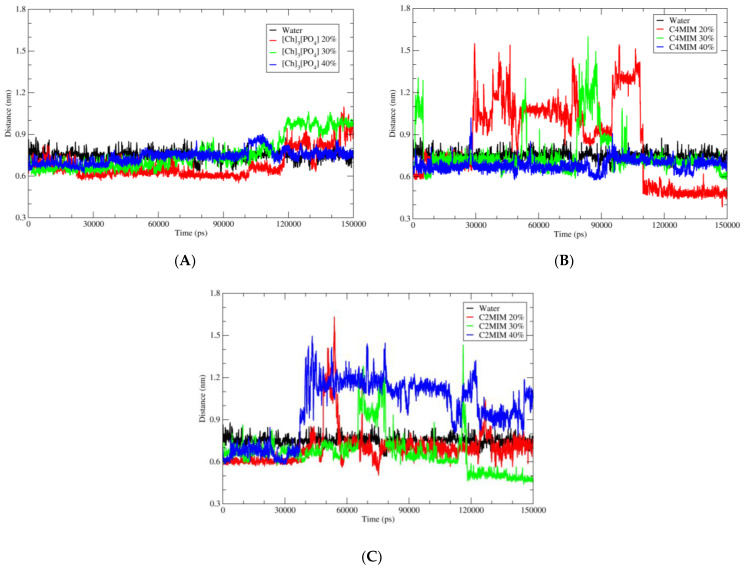
Distance between the C of carbonyl group of BHET with the O of Ser-131 in water and different ionic liquids solutions. (**A**) Pure water in black; 20% of [Ch]_3_[PO_4_] in red; 30% of [Ch]_3_[PO_4_] in green and 40% of [Ch]_3_[PO_4_] in blue. (**B**) Pure water in black; 20% of [C4MIM][Ac] in red; 30% of [C4MIM][Ac] in green and 40% of [C4MIM][Ac] in blue. (**C**) Pure water in black; 20% of [C2MIM][Ac] in red; 30% of [C2MIM][Ac] in green and 40% of [C2MIM][Ac] in blue.

After analyzing the overall structure of PETase, we focused on the active site of PETase, where BHET ligand binds and is degraded into smaller units through the catalytic reaction. The catalytic triad is present on the protein surface. The triad is composed of Serine 131, Histidine 208, and Aspartic acid 177. We investigated the distance between our ligand (BHET) carbonyl carbon and oxygen of Ser-131. In order to understand the stability of the interaction between ligand and catalytic residue, the distance between the ligand and Ser-131 in the presence of water and all ionic liquids in all three ionic concentrations was calculated over the whole duration of MD simulation. As depicted in Figure 7, the distance between these two residues is stable over the simulation in water and in [Ch]_3_[PO_4_] as shown in Figure 7A.

The black curve shows the distance in the water and the blue one with [Ch]_3_[PO_4_] at 40% concentration. This shows the stability of ligand during the simulation; hence the condition that is favorable for the reaction is maintained. Therefore, one can consider [Ch]_3_[PO_4_] ionic liquid as a possible medium for BHET hydrolysis. However, in the case of [C4MIM][Ac] and [C2MIM][Ac], the distance fluctuates significantly during the simulation, which implies the instability of ligand on the active site of PETase. Among the ionic liquids, [Ch]_3_[PO_4_] illustrated the best result for maintaining ligand stability at the catalytic site.

### 2.5. Hydrogen Bonds and Hydrophobic Interaction

The solubility and stability of a protein are also dependent on its solvent interaction. The solvent interaction can stabilize or destabilize the amino acids of the protein. Moreover, the hydrogen bond interaction plays an important role in the overall stability of the protein structure. Thus, the number of H bonds with the protein demonstrates its structural stability.

**Figure 8:**
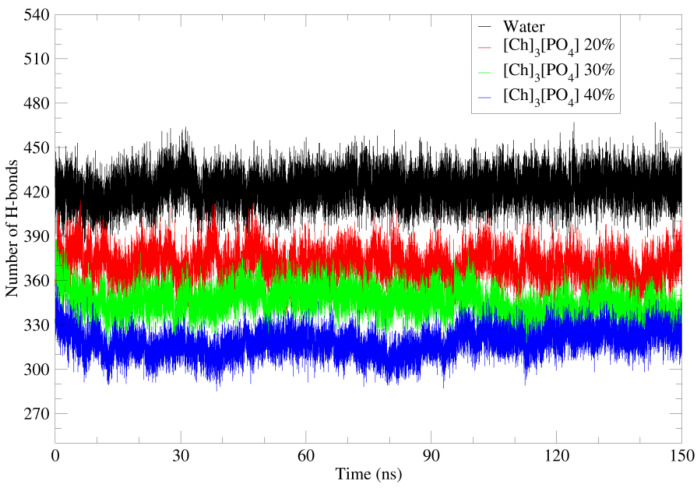
Number of hydrogen bonds of water and cholinium phosphate with amino acids of PETase in 40%, 30%, and 20% IL solutions. Pure water in black; 20% of [Ch]_3_[PO_4_] in red; 30% of [Ch]_3_[PO_4_] in green and 40% of [Ch]_3_[PO_4_] in blue.

As shown in Figure 8, the number of H bonds of water molecules, cholinium, and phosphate ions with protein residues were calculated in different ionic liquid concentrations. Here, 40%, 30%, and 20% concentrations of each system were calculated by adding the solvent values for a separate system.

Figure 8 illustrated that, when pure water was replaced with aqueous solutions of ionic liquids, the number of hydrogen bonds decreased, probably due to the bigger size of cation and anions. Furthermore, the number of H bonds in the 20% solution is higher than the one in the 40% solution. However, in the case of water-[C4MIM][Ac] and water-[C2MIM][Ac] (Supplementary data Figure S1A,B), the [C4MIM] and [C2MIM] cations do not play important role for H bonding with the PETase, as they do not have groups that promote H bonds. However, there is no significant change in H bonds for different concentrations of [C4MIM][Ac] and [C2MIM][Ac].

**Figure 9:**
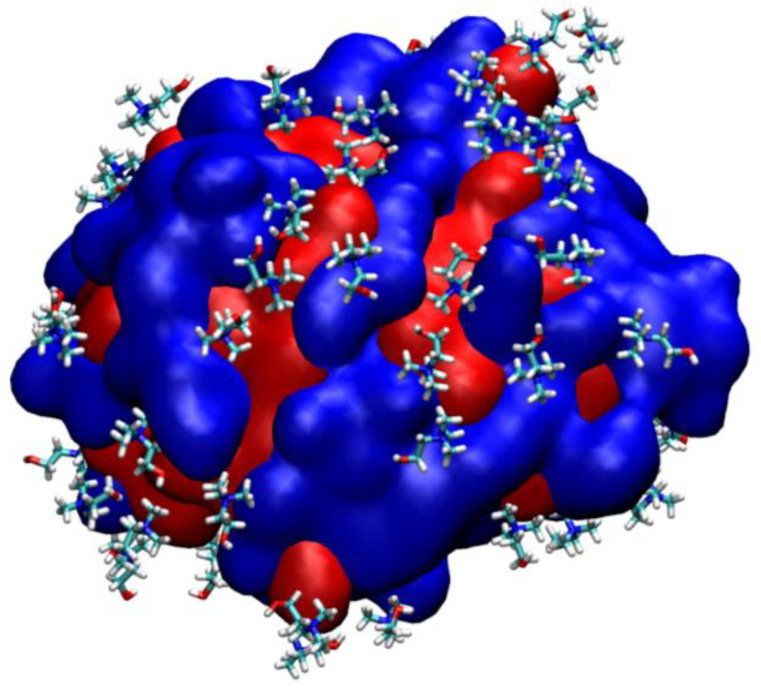
The interaction of cholinium cations with hydrophobic surface of PETase. The hydrophobic surface is colored in red and the hydrophilic surface is colored in blue.

Furthermore, the hydrophobic amino acids are also located at the surface of PETase, which are not solvated by water molecules. Therefore, they interact more with the hydrophobic part of [C4MIM], [C2MIM], and cholinium cations. The hydrophobic interaction took place between hydrophobic amino acids, such as A123, F26, P145, A197, P9, A11, F232, and F162, and methyl groups of cholinium, hydrocarbon (-CH_2_-CH_3_), or the imidazolium ring of [C4MIM] and [C2MIM] cation. Hence, cholinium cations (Figure 9), [C4MIM] and [C2MIM] (Supplementary data Figure S2A,B) are mainly distributed over the hydrophobic surface of PETase.

### 2.6. Radial Distribution Function

The radial distribution function (RDF)—the probability of finding a particle from a reference particle over distance—is a powerful tool in MD simulations for understanding how the (CH3)_3_N^+^ group of cholinium cation, the ring of imidazolium [C4MIM], and [C2MIM] are distributed around the side chain of the hydrophobic surface amino acids.

**Figure 10:**
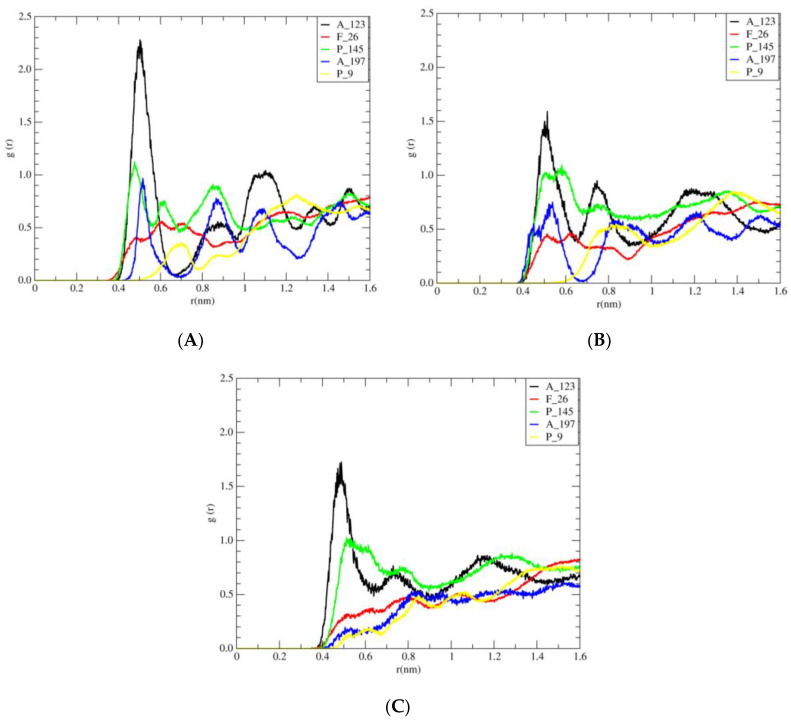
Radial distribution functions (RDFs) for atoms with (**A**) side chain of amino acids A_123 (in black), F_26 (in red), P_145 (in green), A_197 (in blue), and P_9 (in yellow) of the hydrophobic surface and nitrogen of cholinium cations in 40% of [Ch]_3_[PO_4_]. (**B**) Side chain of amino acids A_123, F_26, P_145, A_197, and P_9 of the hydrophobic surface and nitrogen of cholinium cations in 30% of [Ch]_3_[PO_4_]. (**C**) Side chain of amino acids A_123, F_26, P_145, A_197, and P_9 of the hydrophobic surface and nitrogen of cholinium cations in 20% of [Ch]_3_[PO_4_].

The RDF of the (CH_3_)_3_N^+^ group of cholinium cation around the side chain of hydrophobic amino acids at the protein surface (such as A_123, F_26, P_145, A_197, and P_9), the RDF of the ring of [C4MIM] around the side chain of hydrophobic amino acids at the protein surface (such as A_123, A1_1, F_232, P_9, and F_162), and the RDF of the ring of [C2MIM] around side chain of hydrophobic amino acids at the protein surface (such as A_123, A_11, F_232, P_9, and F_162), were calculated, and the results are shown in Figure 10, Supplementary data Figure S3, and Supplementary data Figure S4, respectively.

As shown in Figure 10, in different concentrations (40%, 30%, and 20%), the height of the first peak (A_123) of the distribution function is larger than the rest of the amino acids. According to Figure S3, in different concentrations (40%, 30%, and 20% of [C4MIM][Ac]), the height of the first peak (A_123) in the radial distribution function is greater. As shown in Figure S4, in all designed systems of [C2MIM][Ac] in different concentrations 40%, 30%, and 20%, the height of the first peak (A_123) of the RDF is larger than the rest. Thus, in accordance with Figure 10 through Figure S4, the height of the first peak (A_123) for different ionic liquids is as follows:

Alkyl groups of [C2MIM] cation < Alkyl groups of [C4MIM] cation < methyl groups of cholinium cation.

On the other hand, the highest peak in RDF corresponds to the probability of methyl groups of cholinium cation around the methyl group of A_123 (Figure 10). From Figure 10, can be seen that methyl groups of cation showed insignificant differences of probability density around the rings of F_26 and P_9. As it was expected from A_123 and P145, the probability density of methyl groups of cholinium cation was more for the methyl group (side chain) of A_123 than for the ring of P_145.

Furthermore, by decreasing the concentration of [Ch]_3_[PO_4_], the density of cations around A_123 is also decreased. It can be also concluded that with the increase in the length of the alkyl chain from [C2MIM] to [C4MIM], the density of the cations around A_123 increased, but by increasing the concentration (from 20% to 40%) of [C2MIM][Ac] and [C4MIM][Ac], the change in the density of these cations is not significant. Thus, the effect of crowding on the stability of the hydrophobic surface is more pronounced in the presence of [Ch]_3_[PO_4_] than other ionic liquids.

## 3. Materials and Methods

Several different model systems were set up to perform molecular dynamics simulations of wild-type *Ideonella sakaiensis* PETase enzyme with the PDB code of 5XJH [39]. The simulation systems with three different concentration of three ILs of 1-ethyl-3-methylimidazolium acetate [C2MIM][Ac], 1-butyl-3-methylimidazolium acetate [C4MIM][Ac], and cholinium phosphate (choline phosphate) [Ch]_3_[PO_4_] were prepared. Recent studies reveal that [Ch]_3_[PO_4_] is a bi-functional media, capable of both high solubilization (solvent) and effective depolymerization (catalyst) of PET [4]. Furthermore, [Ch]_3_[PO_4_] is also an effective depolymerizer (catalysts). Furthermore, the lower cost of cholinium phosphate [Ch]_3_[PO_4_] ionic liquid has quite a good ability to dissolve PET (10 wt%) [4]. Hence, the initial structures with these ionic liquids were setup using PACKMOL [40]. As a control, the PETase enzyme was also simulated in pure aqueous media. The ligand presented in our study has two TPA rings, which are called BHET. Parametrization of the ligand and ILs were carried out using general amber force field GAFF [41], as this forcefield can accurately predict thermodynamic and transport properties of various ionic liquids [42], and we have successfully used it in our previous calculations with ILs [43,44]. Each system was placed in a cubic box, sized 12 nm, using PACKMOL program package. Each model system has been neutralized by counterions. The system was first equilibrated in canonical ensemble (NVT) for 500 ps at 300 K temperature followed by the NPT—the isothermal isobaric ensemble. The temperature was maintained at 300 K and the pressure was maintained at 1bar with compressibility of 4.6 × 10^−5^/bar by weak coupling to temperature and pressure baths using the Berendsen method [45] with relaxation times of 0.1 ps. Van der Waals forces were evaluated with a Lennard-Jones potential, having 10 Å cut-off, and long-range electrostatic contributions were evaluated using the particle mesh Ewald method [46] with a direct interaction cut-off of 10.0 Å. A time step of 2 fs was employed. Lengths of all covalent bonds were constrained by the linear constraint solver algorithm (LINCS) [47]. All simulations were run with periodic boundary conditions for 150 ns. The MD simulations and related analyses were performed using the GROMACS program package [48,49,50]. Molecular graphics images were produced using VMD [51] software and all the graphs were prepared using xmgrace program.

## 4. Conclusions

In the current study, the structural basis of the possible co-solvent candidates for the stability of PETase were analyzed and their interaction patterns were compared. The in silico-based investigation, using MD simulations, provides new insights into the interacting residues of the protein with the ionic liquids and water. The effect of ionic liquids cholinium phosphate [Ch]_3_[PO_4_], 1-butyl-3-methyl- imidazolium acetate [C4MIM][Ac], 1-ethyl-3-methyl-imidazolium acetate [C2MIM][Ac], and their water solutions on *Ideonella sakaiensis* PETase with BHET substrate were investigated. Moreover, after the careful structural and interactive analyses of the ligand with the PETase, it is concluded that among all the above mentioned ionic liquids, [Ch]_3_[PO_4_] has the smallest effect of the structure of PETase and its interaction with the BHET substrate, which is comparable to the pure water solvent. This makes the water solution with [Ch]_3_[PO_4_] a good candidate for the solvent used during BHET hydrolysis, which can be used for future investigation.

## Data Availability

The data presented in this study are available in article.

## Supplementary Materials

The following supporting information can be downloaded online, Figure S1: The number of hydrogen bonds between amino acids of PETase and [C2MIM][Ac], [C4MIM][Ac]; Figure S2: the interaction of [C4MIM] (A) and [C2MIM] (B) cations with hydrophobic surface of PETase; Figure S3: radial distribution functions (RDFs) for atoms of hydrophobic amino acids and [C4MIM] cations; Figure S4: additionally, the radial distribution functions (RDFs) for atoms of hydrophobic amino acids and [C2MIM] cations are included in the supplementary data file.

## References

[1] Cimpan C., Bjelle E.L., Stromman A.H. (2021). Plastic packaging flows in Europe: A hybrid input-output approach. J. Ind. Ecol..

[2] Rochman C.M., Browne M.A., Halpern B.S., Hentschel B.T., Hoh E., Karapanagioti H.K., Rios-Mendoza L.M., Takada H., Teh S., Thompson R.C. (2013). Classify plastic waste as hazardous. Nature.

[3] Geyer R., Jambeck J.R., Law K.L. (2017). Production, use, and fate of all plastics ever made. Sci. Adv..

[4] Sun J., Liu D., Young R.P., Cruz A.G., Isern N.G., Schuerg T., Cort J.R., Simmons B.A., Singh S. (2018). Solubilization and Upgrading of High Polyethylene Terephthalate Loadings in a Low-Costing Bifunctional Ionic Liquid. ChemSusChem.

[5] Todd A.D., McEneany R.J., Topolkaraev V.A., Macosko C.W., Hillmyer M.A. (2016). Reactive compatibilization of poly (ethylene terephthalate) and high-density polyethylene using amino-telechelic polyethylene. Macromolecules.

[6] Shi H., Tang A., Liang Q., Jiang Y. (2016). Synthesis and hydrophobic properties of F & Si containing poly (ethylene terephthalate). RSC Adv..

[7] Wang H., Li Z., Liu Y., Zhang X., Zhang S. (2009). Degradation of poly (ethylene terephthalate) using ionic liquids. Green Chem..

[8] Chaudhary N., Koiry S., Singh A., Tillu A., Jha P., Samanta S., Debnath A., Aswal D., Mondal R., Acharya S. (2017). Electron beam induced modifications in flexible biaxially oriented polyethylene terephthalate sheets: Improved mechanical and electrical properties. Mater. Chem. Phys..

[9] Samak N.A., Jia Y., Sharshar M.M., Mu T., Yang M., Peh S., Xing J. (2020). Recent advances in biocatalysts engineering for polyethylene terephthalate plastic waste green recycling. Environ. Int..

[10] Wallace N.E., Adams M.C., Chafin A.C., Jones D.D., Tsui C.L., Gruber T.D. (2020). The highly crystalline PET found in plastic water bottles does not support the growth of the PETase-producing bacterium Ideonella sakaiensis. Environ. Microbiol. Rep..

[11] Olivier-Bourbigou H., Magna L., Morvan D. (2010). Ionic liquids and catalysis: Recent progress from knowledge to applications. Appl. Catal. A Gen..

[12] Moniruzzaman M., Nakashima K., Kamiya N., Goto M. (2010). Recent advances of enzymatic reactions in ionic liquids. Biochem. Eng. J..

[13] Hadad C., Husson E., Van Nhien A.N. (2020). Conversion of Chitin in Ionic Liquids. Encyclopedia of Ionic Liquids.

[14] Bubalo M.C., Radošević K., Redovniković I.R., Slivac I., Srček V.G. (2017). Toxicity mechanisms of ionic liquids. Arch. Ind. Hyg. Toxicol..

[15] Roosen C., Müller P., Greiner L. (2008). Ionic liquids in biotechnology: Applications and perspectives for biotransformations. Appl. Microbiol. Biotechnol..

[16] Gorke J., Srienc F., Kazlauskas R. (2010). Toward advanced ionic liquids. Polar, enzyme-friendly solvents for biocatalysis. Biotechnol. Bioprocess Eng..

[17] Halle B. (2004). Protein hydration dynamics in solution: A critical survey. Philos. Trans. R. Soc. Lond. Ser. B Biol. Sci..

[18] Klibanov A.M. (2001). Improving enzymes by using them in organic solvents. Nature.

[19] Constatinescu D., Herrmann C., Weingärtner H. (2010). Patterns of protein unfolding and protein aggregation in ionic liquids. PCCP.

[20] Page T.A., Kraut N.D., Page P.M., Baker G.A., Bright F.V. (2009). Dynamics of loop 1 of domain I in human serum albumin when dissolved in ionic liquids. J. Phys. Chem. B.

[21] Akdogan Y., Junk M.J., Hinderberger D. (2011). Effect of ionic liquids on the solution structure of human serum albumin. Biomacromolecules.

[22] Micaelo N.M., Soares C.M. (2008). Protein structure and dynamics in ionic liquids. Insights from molecular dynamics simulation studies. J. Phys. Chem. B.

[23] Klähn M., Lim G.S., Seduraman A., Wu P. (2011). On the different roles of anions and cations in the solvation of enzymes in ionic liquids. PCCP.

[24] Wang H., Gurau G., Rogers R.D. (2012). Ionic liquid processing of cellulose. Chem. Soc. Rev..

[25] Xu A.R., Wang F. (2020). Carboxylate ionic liquid solvent systems from 2006 to 2020: Thermal properties and application in cellulose processing. Green Chem..

[26] Tournier V., Topham C., Gilles A., David B., Folgoas C., Moya-Leclair E., Kamionka E., Desrousseaux M.-L., Texier H., Gavalda S. (2020). An engineered PET depolymerase to break down and recycle plastic bottles. Nature.

[27] Müller R.J., Schrader H., Profe J., Dresler K., Deckwer W.D. (2005). Enzymatic degradation of poly (ethylene terephthalate): Rapid hydrolyse using a hydrolase from T. fusca. Macromol. Rapid Commun..

[28] Sinha V., Patel M.R., Patel J.V. (2010). PET waste management by chemical recycling: A review. J. Polym. Environ..

[29] Liu B., He L., Wang L., Li T., Li C., Liu H., Luo Y., Bao R. (2018). Protein crystallography and site-direct mutagenesis analysis of the poly (ethylene terephthalate) hydrolase PETase from Ideonella sakaiensis. ChemBioChem.

[30] Yang Y., Yang J., Jiang L. (2016). Comment on “A bacterium that degrades and assimilates poly (ethylene terephthalate)”. Science.

[31] Yoshida S., Hiraga K., Takehana T., Taniguchi I., Yamaji H., Maeda Y., Toyohara K., Miyamoto K., Kimura Y., Oda K. (2016). A bacterium that degrades and assimilates poly (ethylene terephthalate). Science.

[32] Bornscheuer U.T. (2016). Feeding on plastic. Science.

[33] Han X., Liu W., Huang J.-W., Ma J., Zheng Y., Ko T.-P., Xu L., Cheng Y.-S., Chen C.-C., Guo R.-T. (2017). Structural insight into catalytic mechanism of PET hydrolase. Nat. Commun..

[34] Palm G.J., Reisky L., Böttcher D., Müller H., Michels E.A., Walczak M.C., Berndt L., Weiss M.S., Bornscheuer U.T., Weber G. (2019). Structure of the plastic-degrading Ideonella sakaiensis MHETase bound to a substrate. Nat. Commun..

[35] Marshall I., Todd A. (1953). The thermal degradation of polyethylene terephthalate. Trans. Faraday Soc..

[36] Tokiwa Y., Calabia B.P., Ugwu C.U., Aiba S. (2009). Biodegradability of plastics. Int. J. Mol. Sci..

[37] Austin H.P., Allen M.D., Donohoe B.S., Rorrer N.A., Kearns F.L., Silveira R.L., Pollard B.C., Dominick G., Duman R., El Omari K. (2018). Characterization and engineering of a plastic-degrading aromatic polyesterase. Proc. Natl. Acad. Sci. USA.

[38] Baldwin R.L. (1996). How Hofmeister ion interactions affect protein stability. Biophys. J..

[39] Joo S., Cho I.J., Seo H., Son H.F., Sagong H.-Y., Shin T.J., Choi S.Y., Lee S.Y., Kim K.-J. (2018). Structural insight into molecular mechanism of poly (ethylene terephthalate) degradation. Nat. Commun..

[40] Martínez L., Andrade R., Birgin E.G., Martínez J.M. (2009). PACKMOL: A package for building initial configurations for molecular dynamics simulations. J. Comput. Chem..

[41] Dickson C.J., Madej B.D., Skjevik Å.A., Betz R.M., Teigen K., Gould I.R., Walker R.C. (2014). Lipid14: The amber lipid force field. J. Chem. Theory Comput..

[42] Sprenger K.G., Jaeger V.W., Pfaendtner J. (2015). The General AMBER Force Field (GAFF) Can Accurately Predict Thermodynamic and Transport Properties of Many Ionic Liquids. J. Phys. Chem. B.

[43] D’Oronzo E., Secundo F., Minofar B., Kulik N., Pometun A.A., Tishkov V.I. (2018). Activation/Inactivation Role of Ionic Liquids on Formate Dehydrogenase from Pseudomonas sp 101 and Its Mutated Thermostable Form. Chemcatchem.

[44] Shaposhnikova A., Kuty M., Chaloupkova R., Damborsky J., Smatanova I.K., Minofar B., Prudnikova T. (2021). Stabilization of Haloalkane Dehalogenase Structure by Interfacial Interaction with Ionic Liquids. Crystals.

[45] Ryckaert J.-P., Ciccotti G., Berendsen H.J. (1977). Numerical integration of the cartesian equations of motion of a system with constraints: Molecular dynamics of n-alkanes. J. Comput. Phys..

[46] Essmann U., Perera L., Berkowitz M.L., Darden T., Lee H., Pedersen L.G. (1995). A smooth particle mesh Ewald method. J. Chem. Phys..

[47] Hess B. (2008). P-LINCS: A parallel linear constraint solver for molecular simulation. J. Chem. Theory Comput..

[48] Berendsen H.J.C., Vanderspoel D., Vandrunen R. (1995). GROMACS—A message-passing parallel molecular-dynamics implementation. Comput. Phys. Commun..

[49] Pronk S., Pall S., Schulz R., Larsson P., Bjelkmar P., Apostolov R., Shirts M.R., Smith J.C., Kasson P.M., van der Spoel D. (2013). GROMACS 4.5: A high-throughput and highly parallel open source molecular simulation toolkit. Bioinformatics.

[50] Van der Spoel D., Lindahl E., Hess B., Groenhof G., Mark A.E., Berendsen H.J.C. (2005). GROMACS: Fast, flexible, and free. J. Comput. Chem..

[51] Humphrey W., Dalke A., Schulten K. (1996). VMD: Visual molecular dynamics. J. Mol. Graph..

