# Supplementary material for: Surface Interaction of Ionic Liquids: Stabilization of Polyethylene Terephthalate-Degrading Enzymes in Solution
Source: Molecules. 2021 Dec 26;27(1):119. doi: 10.3390/molecules27010119 (PMC8746539; doi:10.3390/molecules27010119)
Supplement: Supplementary file 1 [file molecules-27-00119-s001.zip › molecules-1511218-SM.pdf]

Supplementary date

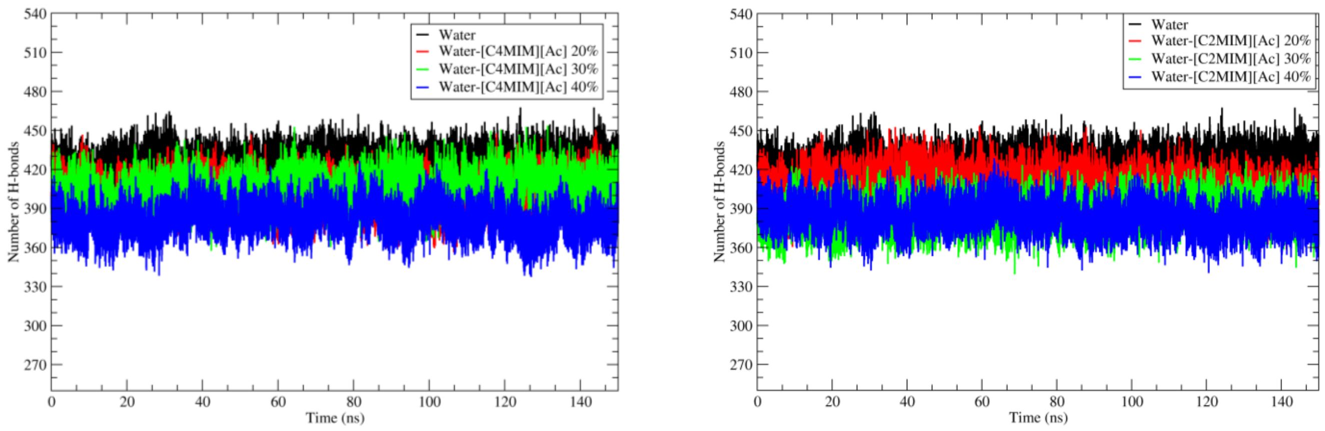

(**A**) (**B**)

Figure S1. Number of hydrogen bonds between amino acids of PETase and solvents. (**A**); pure water in black, in red 20% of solvent Water-[C4MIM][Ac], in green 30% of solvent Water-[C4MIM][Ac] and in blue 40% of solvent Water-[C4MIM][Ac]. (**B**); pure water in black, in red 20% of solvent Water-[C2MIM][Ac], in green 30% of solvent Water-[C2MIM][Ac] and in blue 40% of solvent Water-[C2MIM][Ac].

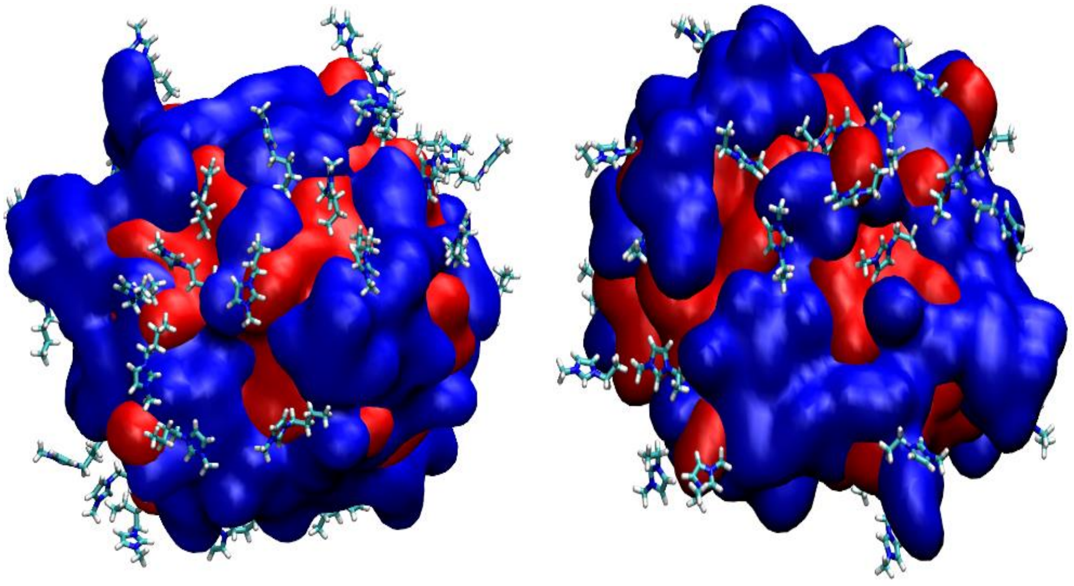

(**A**) (**B**)

Figure S2. The interaction of [C4MIM] (**A**) and [C2MIM] (**B**) cations with hydrophobic surface of PETase. The hydrophobic surface is colored in red and the hydrophilic surface is colored in blue.

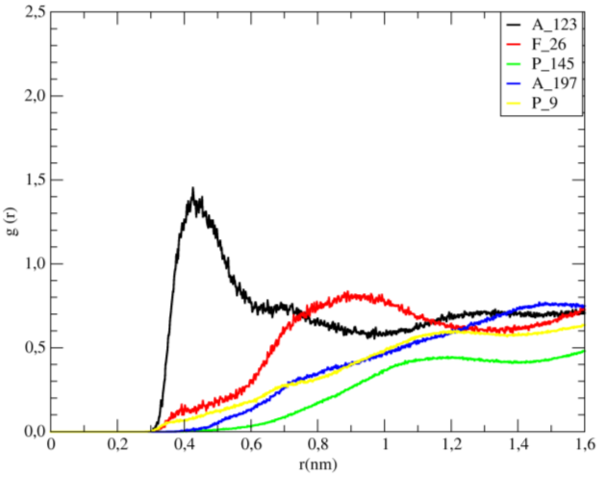

(**A**)

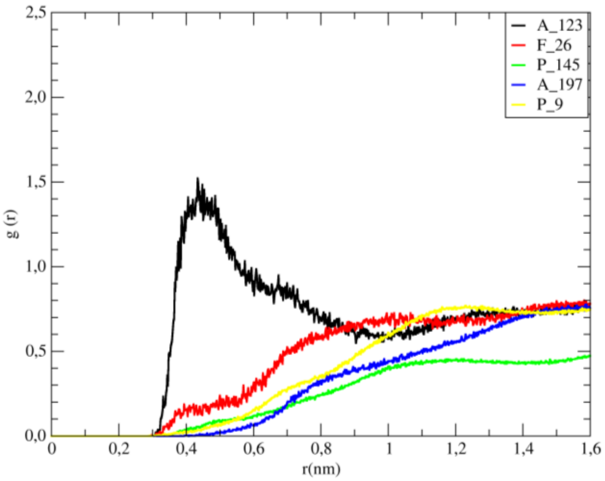

(**B**)

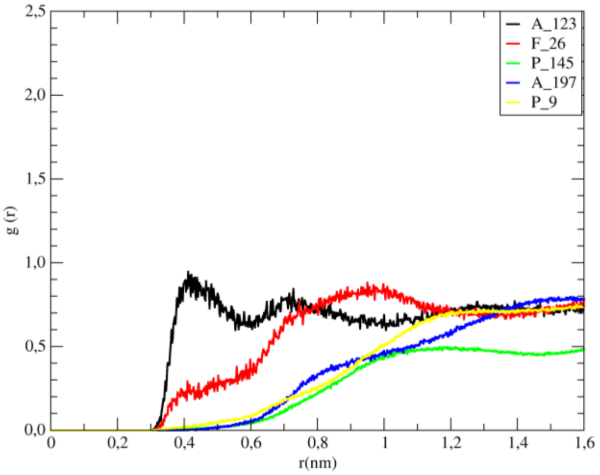

(**C**)

Figure S3. Radia distribution functions (RDFs) for atoms with (**A**) side chain of amino acids A123 (in black), A11 (in red), F232 (in green), P9 (in blue) and F162 (in yellow) of hydrophobic surface of PETase and ring of [C4MIM] in 40% of [C4MIM][Ac]. (**B**); side chain of amino acids A123, A11, F232, P9 and F162 of hydrophobic surface of PETase and ring of [C4MIM] in 30% of [C4MIM][Ac]. (**C**); side chain of amino acids A123, A11, F232, P9 and F162 of hydrophobic surface of PETase and ring of [C4MIM] in 20% of [C4MIM][Ac].

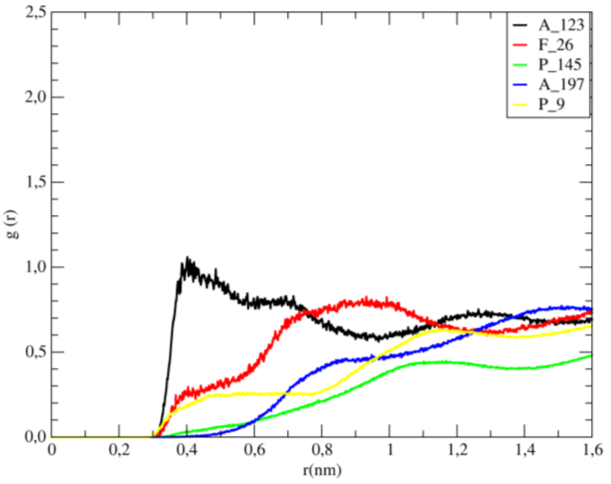

(**A**)

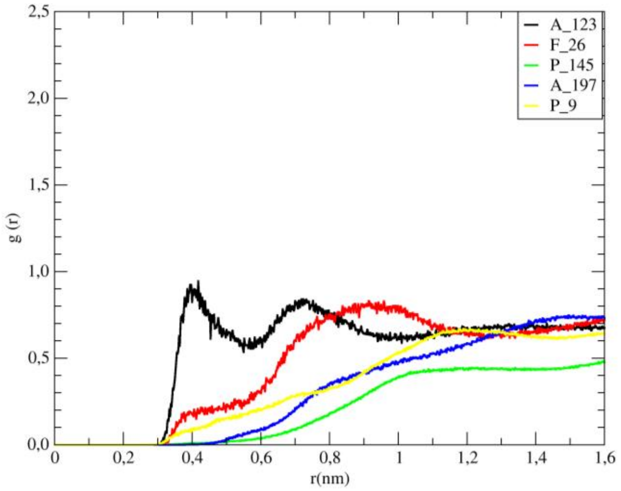

(**B**)

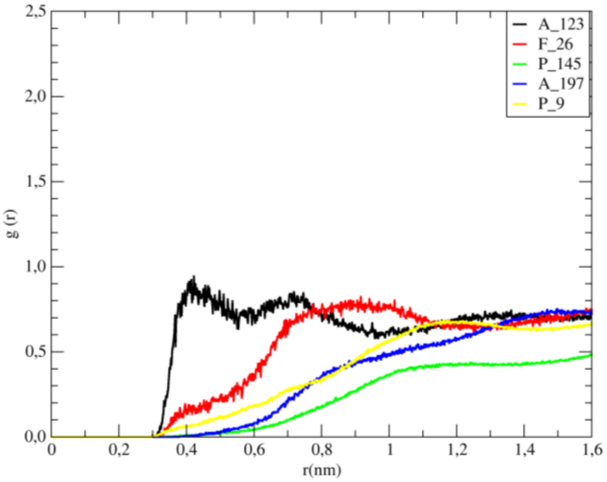

(**C**)

Figure S4. Radia distribution functions (RDFs) for atoms with (**A**) side chain of amino acids A123 (in black), A11 (in red), F232 (in green), P9 (in blue) and F162 (in yellow) of hydrophobic surface of PETase and ring of [C2MIM] in 40% of [C2MIM][Ac]. (**B**); side chain of amino acids A123, A11, F232, P9 and F162 of hydrophobic surface of PETase and ring of [C2MIM] in 30% of [C2MIM][Ac]. (**C**); side chain of amino acids A123, A11, F232, P9 and F162 of hydrophobic surface of PETase and ring of [C2MIM] in 20% of [C2MIM][Ac].
